# An unexpected, pH-sensitive step of the enterovirus D68 lifecycle

**DOI:** 10.1128/mbio.02281-23

**Published:** 2023-11-01

**Authors:** David Aponte-Diaz, Matthew R. Vogt, Craig E. Cameron

**Affiliations:** 1 Department of Microbiology and Immunology, The University of North Carolina at Chapel Hill, Chapel Hill, North Carolina, USA; 2 Department of Pediatrics, Division of Infectious Diseases, The University of North Carolina at Chapel Hill, Chapel Hill, North Carolina, USA; Duke University School of Medicine, Durham, North Carolina, USA

**Keywords:** enterovirus, poliovirus, picornavirus

## Abstract

Enterovirus D68 (EV-D68) contributes significantly to pathogen-induced respiratory illnesses and severe neurological disorders like acute flaccid myelitis. We lack EV-D68 preventive measures, and knowledge of its molecular and cellular biology is incomplete. Multiple studies have highlighted the role of membrane compartments and autophagy during picornavirus multiplication. Galitska et al. found that EV-D68 also exploits cellular autophagic compartments and relies on autophagic machinery as pro-viral factors (G. Galitska, A. Jassey, M. A. Wagner, N. Pollack, et al., mBio e02141-23, 2023, https://doi.org/10.1128/mbio.02141-23). Surprisingly, failure of the autophagic compartment to acidify early during EV-D68 infection causes a delay in RNA synthesis that has not been reported for other enteroviruses. This delay appears to reflect the inability of viral proteins 2B and 3A to engage membranes stably, leading to their degradation in the cytoplasm. Observations like this underscore the importance of studying individual members of the virus genus. It will be interesting to understand how this phenomenon connects to EV-D68 pathogenesis, if at all.

## COMMENTARY

Picornaviruses are responsible for nearly half of all acute respiratory illness (ARI) cases caused by viral pathogens (1). Enterovirus D68 (EV-D68), an emerging respiratory picornavirus, manifests various illnesses, from typical ARI to severe but reversible wheezing illness to less common yet permanent paralysis (2, 3). This paralyzing syndrome is acute flaccid myelitis (AFM), with recent outbreaks driven by EV-D68 infections. AFM caused by EV-D68 is clinically indistinguishable from poliomyelitis, caused by the closely related picornavirus poliovirus (PV) (4). While they share this paralytic endpoint, EV-D68 infections typically begin in the respiratory tract, whereas PV infects the gastrointestinal mucosa. Further differentiating these viruses, unlike PV, no vaccines or therapeutics are currently available to prevent EV-D68 infection or treat its associated diseases (5). In response to this pressing need, picornavirologists have directed their research efforts toward comprehending the molecular mechanisms governing EV-D68 multiplication and pathogenesis, often utilizing PV and other well-established enteroviruses as models for comparison.

**Fig 1:**
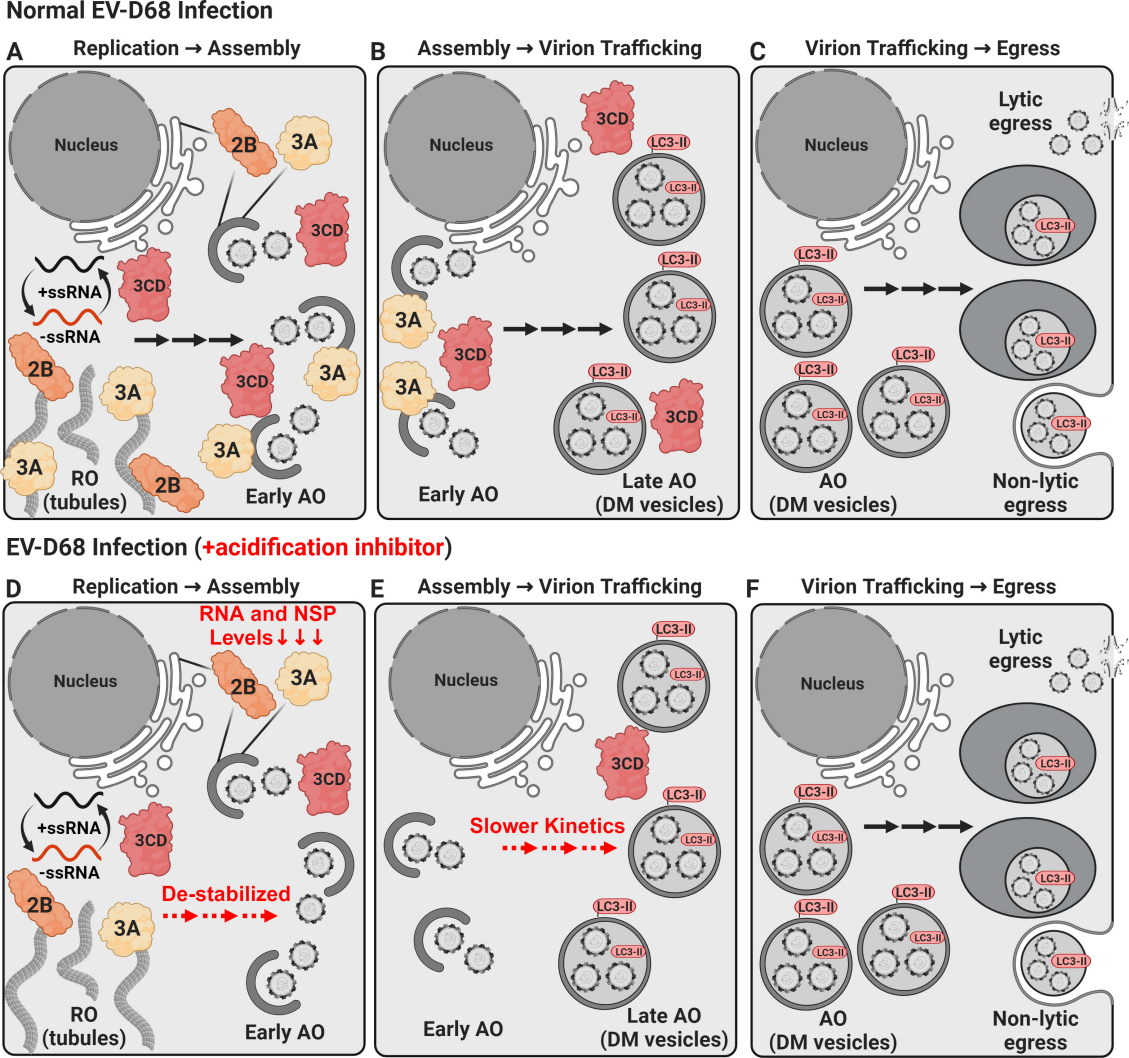
Impact of acidification inhibitors at different stages of the EV-D68 lifecycle. (**A**) Replication organelle (RO) formation: early during infection, tubular replication organelles form and serve as the site of genome replication. The viral 3CD protein is required for the genesis of the RO and interacts with phosphoinositides, decorating the RO (8, 15, 16). Several membrane-associated viral proteins interact with the RO, specifically proteins 2B and 3A (17). As the infection progresses, these tubules transform into structures resembling omegasomes, the early assembly organelle (AO). (**B**) Assembly organelle: the late assembly organelle is characterized by double-membrane (DM) structures of autophagic origin decorated by lipidated LC3 protein (LC3-II). Virus particles are enclosed in these compartments. (**C**) Virus-containing amphisome production: double-membrane structures fuse with endosomes and/or multivesicular bodies, forming virus-containing amphisomes. (**D**) Impact of acidification inhibitors: failure of the RO/AO compartment to acidify appears to destabilize proteins 2B and 3A, perhaps by altering their association with membranes and leading to their degradation in the cytoplasm. PV 2B and 3A do not exhibit this pH sensitivity. (**E**) Reduced levels of 2B and 3A only reduce the rate of virus production. (**F**) Total virus yield remains normal, likely reflecting the high multiplicity of infection (MOI) used. It is known that enterovirus fecundity diminishes as a function of MOI (8). Created with BioRender.com.

Enteroviruses replicate their genome in association with cellular membranes (Fig. 1A) (6, 7). These membranes and membranous compartments also play critical roles in the post-replication stages of the virus lifecycle (Fig. 1B and C) (8). Pioneering work by Kirkegaard and other researchers has established a role of macroautophagy (a cellular pathway for waste degradation and recycling) in facilitating enterovirus multiplication within host cells (9, 10). Recent studies by Jackson and others have indicated that PV infection relies on autophagy-derived acidic compartments for virion morphogenesis (11, 12). In their latest study, Galitska and colleagues sought to determine if EV-D68 exploits the cellular autophagic machinery for virus replication and dissemination (13), drawing comparisons and contrasts with the established mechanisms used by PV to hijack autophagy (12, 14).

In their recent article, Galitska et al. reveal that cellular autophagic compartments play a significant role in EV-D68 virus production, mirroring earlier findings by their group and others in the context of PV (13). They propose that both PV and EV-D68 co-opt the cellular autophagic machinery to yield viable virus progeny. Furthermore, for efficient virus multiplication, both viruses depend on late autophagic membrane-shaping machinery crucial for microtubule-associated protein 1A/1B-light chain 3 (LC3) protein lipidation and autophagosome maturation. The authors present various outcomes of EV-D68 infection following inhibition of acidification of the autophagy-induced membranous compartment. Notably, when an acidification inhibitor is applied, the virus production rate during EV-D68 infection is decreased (slower virus production kinetics) (Fig. 1E) with no impact on final virus yield (Fig. 1F). Conversely, PV infection reduces virus yield (total amount of virus produced at infection endpoint). The initial 30 minutes of viral entry into the cell is associated with a delay in EV-D68 genome synthesis, a phenomenon possibly linked to virus fusion during endocytosis, supported by numerous studies emphasizing the significance of acidification in EV-D68 uncoating (18, 19).

Interestingly, during EV-D68 infection, the levels of non-structural proteins 2B and 3A decrease in the presence of acidification inhibitors, a phenomenon not observed in PV infection under comparable conditions (Fig. 1D). This unique response of EV-D68 is most consistent with the degradation of 2B and 3A proteins. These proteins are known to have a stable interaction with virus-induced membranes. However, the physical basis for these proteins interacting with membranes has not been investigated. The study by Galitska et al. suggests that the intraluminal pH matters for recruitment, association, and/or retention of 2B and 3A in virus-induced membranes. Failure to acidify the compartment leads to a diminution in the formation of the RO and a corresponding reduction in levels of EV-D68 viral RNA (vRNA) (Fig. 1D). It remains unclear whether this effect is due to inhibited genome replication or compromised protection of vRNAs caused by perturbations to the RO, as indicated by their ultrastructural analysis of infected cells. The authors favor the latter. The requirement of low pH for EV-D68 genome replication did not extrapolate to a corresponding reduction in virus yield. This may reflect the high multiplicity of infection used. High MOI may suppress the impact on virus yield. There is no doubt that their future studies will further clarify this unique requirement of EV-D68 for low pH to form the RO and may uncover how this requirement contributes to pathogenesis. The consequences for genome replication when acidification is compromised may lead to new therapeutic interventions for EV-D68 not predicted by studies of PV.
